# The Influence Mechanism of Supervisor Developmental Feedback on the Enactment of Employees’ Creative Ideas: A Moderated Chain Mediation Model Based on Psychological Empowerment

**DOI:** 10.3389/fpsyg.2021.696034

**Published:** 2021-10-21

**Authors:** Haiman Liu, Jiancheng Long

**Affiliations:** School of Economics and Management, Xidian University, Xi'an, China

**Keywords:** supervisor developmental feedback, positive emotions, work engagement, psychological empowerment, enactment of employees’ creative ideas

## Abstract

Employees’ creative idea enactment is critical for organizational creativity assessment and innovation implementation. In the paper, we want to develop and verify a moderated chain mediation model to explore the impact of supervisor developmental feedback on the enactment of employees’ creative ideas, and to investigate the moderating role of psychological empowerment further. Hierarchical regression analyses of the multi-time data from 375 employees in China indicate that positive emotions and work engagement, respectively mediate the relationship between supervisor developmental feedback and employees’ creative idea enactment. Simultaneously, positive emotions and work engagement form chain mediation between supervisor developmental feedback and creative idea enactment. Besides, we find that psychological empowerment negatively moderates the relationship between supervisor developmental feedback and employees’ positive emotions, as well as moderates the chain mediating effect of this paper. The present study not only contributes to the literature on feedback and innovation, but also provides practical guidance on how to seek remedies to facilitate employees’ creative idea enactment from the perspective of human resource management.

## Introduction

In the hypercompetitive environment, creative idea, as the hallmarks of contemporary business, is regarded as the significant source of competitive advantage (Sachpazidu-Wojcicka, 2017; Li et al., 2019). Nevertheless, employees judge whether an idea is creative or novel is subjective, and if creative ideas are not appraised or appreciated by supervisors, the organization’s innovation would lose the opportunity to be implemented (Harvey, 2014; Mueller et al., 2017). Faced with this situation, recent documents have proposed the concept of creative idea enactment, which not only could reflect the novelty, uniqueness, and value of ideas to achieve greater mutual understanding and recognition, but also promote the supervisor’s assessment of creativity and trigger the implementation of innovative decisions (Lu et al., 2019). Idea enactment refers to the vivid illustration of abstract creative ideas in more tangible forms using PowerPoint presentations, demos, or other physical objects, such as animating boards, mockups, drawings, and experimental simulations (Lu et al., 2019). Different from the connotation of creativity (Amabile et al., 2005; Liu et al., 2020), the enactment of creative ideas focuses on the practicality and feasibility of creative ideas (Harvey, 2014; Lee and Farh, 2019; Lu et al., 2019). Therefore, owing to creative idea enactment closely relates to the supervisor’s assessment of creativity and the core competitiveness of the company, it is imperative to seek remedies to accelerate the enactment of employees’ creative ideas in the context of practitioners lamenting the slow pace of organizational innovation.

Creative idea enactment not only requires intense cognitive, psychological, and physical efforts on the part of the individual, but also expects circumstantial conditions conducive to innovation (Knippenberg and Hirst, 2020). As a key resource of the company, employees are faced with pervasive uncertainty during the period of globalization countercurrent and epidemic control. Hence, human resource management (HRM) practitioners place enduring importance on suggestions that encourage employees to obtain feedback from supervisors, which furnish employees with pivotal information on how to effectively perform their duties or perceive threats (Harrison and Dossinger, 2017; Bak, 2020; Kim and Kim, 2020). In literature, scholars demonstrated that the supervisor developmental feedback could affect subordinate task performance (Zheng et al., 2015), innovative behavior (Su et al., 2019; Li et al., 2021), and employee voice (Zhang et al., 2019). Obviously, the existing literature has paid insufficient attention to the catalyzing factors of the enactment of creative ideas (Lu et al., 2019), let alone the relationship between supervisor developmental feedback and employees’ creative idea enactment. Hence, considering the pivotal role of feedback and idea enactment in organizational practice and a scarcity of relevant literature (Harvey, 2014; Lu et al., 2019), this study attempts to replenish the previous studies by clarifying the psychological process by which supervisor developmental feedback influences the enactment of employees’ creative ideas.

Returning to the Chinese context, the supervisor developmental feedback is bound to penetrate into the behavior of employees’ creative idea enactment. In China, the communication methods of supervisor are relatively subtle, and supervisor rarely directly provide feedback valence, such as praise or criticism (Zhang et al., 2017), while as a form of unclear expression of feedback valence, supervisor developmental feedback has been favored by managers in China (Zheng et al., 2015). Generally speaking, supervisor developmental feedback is a form of feedback that stimulates subordinates’ work attitudes and behaviors. It not only effectively boosts the improvement of employees’ intrinsic motivation to irritate their interest in the task itself, but also provides employees with directions for future learning and innovation (Li et al., 2021). In other words, unlike the traditional feedback that only provides employees’ past working behaviors or results, the constructive (as opposed to evaluative or threatening), behavioral focused, and learning-oriented developmental feedback from supervisors is more likely to promote employees to ameliorate or break through their existing jobs (Su et al., 2019), thus facilitating the enactment of creative ideas. It is worth emphasizing that insights of Harvey (2014) on boosting team creativity proposed that enacting ideas by producing physical objects could further aid creative synthesis, and enacting ideas is usually regarded as an implementation activity that occurs at a later stage of the innovation process. Therefore, it is reasonably believed that the enactment of employees’ creative ideas could be promoted and driven by supervisor developmental feedback.

Understanding the psychological process of the enactment of employees’ creative ideas is a matter of great concern. It has been corroborated by previous studies that the affective events theory (AET) is utilized to interpret how workplace events affect employees’ job-related attitudes and behavior through emotions (Weiss and Cropanzano, 1996; Madrid et al., 2019). Based on this logic, supervisor developmental feedback cultivates a creativity-supportive content, and this kind of work-related identification shapes the positive emotions of employees, such as enthusiasm, excitement, and alertness (Zhang et al., 2019). According to the Broaden-and-Build Theory (BBT), individual positive emotions maintain their creative behavior and prosocial actions by expanding one’s momentary thought-action repertoires and psychological resources (Amabile et al., 2005; George and Zhou, 2007). At the same time, Eva et al. (2019) identify that supervisor developmental feedback as also a valuable resource, which helps employees adjust their behavior in line with organizational expectations and recommendations, thereby enhancing their work engagement level. While employees with high levels of work engagement have the psychological flexibility to generate novel ideas (Orth and Volmer, 2017; Maden-Eyiusta, 2021), and perform well in extensive information search and problem-solving (Bhatnagar, 2012), which would be instrumental in boosting the enactment of creative ideas. In addition, psychological literature has found that positive emotions capture a series of personal characteristics, such as low stress, high life satisfaction, and better mental health (Fredrickson et al., 2008), which would guide people to effectively adapt and adjust their thinking patterns to cope more successfully with stress and obstructive events (Conner and Silvia, 2015). In practical work, individuals with high positive emotions have the enthusiasm, vitality, and nerve to overcome obstacles at work, and then emerge higher work engagement (Ouweneel et al., 2012; Gloria and Steinhardt, 2017; Buric and Macuka, 2018). Therefore, the present paper proposes a chain mediation model to test the positive influence of supervisor developmental feedback on employees’ creative idea enactment through positive emotions and work engagement.

However, the process of employees’ emotional responses and subsequent behavior triggered by workplace events would be disturbed by personal characteristics (Weiss and Cropanzano, 1996), while psychological empowerment is a psychological state that exists within individuals, reflecting a positive orientation towards job roles (Thomas and Velthouse, 1990). Individuals with different levels of psychological empowerment have inconsistent demands for external resources and knowledge (Audenaert et al., 2017; Kang et al., 2020), leading them to perceive different intensities of emotions or other psychological responses from feedback. For instance, Hong and Gajendran (2018) suggest that psychological empowerment moderates the relationship between certain organizational variables and employee behavior, which makes the hypothetical development of our paper traceable. Thus, we regard psychological empowerment as a psychological state which may be relatively independent of supervisor feedback, and it interferes with the positive emotion intensity and the related chained mediating effect in the relationship between supervisor development feedback and the enactment of employees’ creative ideas.

With the work above, we have summarized several possible marginal contributions. Firstly, by assessing the relationship between supervisor developmental feedback and creative idea enactment through establishing a multi-level theoretical model, the present article seeks to address theoretical gaps and advance the extant knowledge. Using social exchange theory and the Pygmalion effect (Zhou and George, 2001), we deduce for the first time a concise and coherent theoretical explanation of the connection between supervisor development feedback and creative idea enactment. Social exchange theory is considered to interpret the resource-feedback processes during which recipients may implement constructive improvements by absorbing more momentous feedback from their supervisors (Cropanzano et al., 2017; Eva et al., 2019). The meaning of the Pygmalion effect in psychology is that the higher the expectations of people, the better they perform (Szumski and Karwowski, 2019). According to this logic, supervisor developmental feedback reflects the supervisor’s expectations of subordinates, while in turn, employees’ motivation for experimentations and creative attempts would be enhanced according to the principle of reciprocity (Zhang et al., 2017). The pioneering study would deepen the understanding of the emergence and propagation of supervisor developmental feedback. Secondly, in terms of research content, we provide a research framework of the “main effect test – chain mediation test.” This framework emphasizes the psychological process in which the supervisor development feedback influences the enactment of creative ideas, that is, positive emotions and work engagement play a chained mediating role between the two. Lastly, this paper deepens our knowledge of the moderating role of psychological empowerment by testing the extent to which it moderates positive emotional response to supervisor feedback and testing whether it moderates the chained mediating effect we proposed. This article aims to provide theoretical reference and practical enlightenment for how to facilitate employees’ creative idea enactment. Based on the above analysis, we generate the following four questions (Qs):

Q1: How does the supervisor developmental feedback affect the enactment of employees’ creative ideas?
Q2: How do positive emotions and job engagement, respectively, interfere with the relationship between supervisor developmental feedback and the enactment of employees’ creative ideas?
Q3: Do positive emotions and work engagement play a chained mediating role in the influence of supervisor developmental feedback on employees’ creative idea enactment?
Q4: How does psychological empowerment interfere with the complex influence mechanism of supervisor developmental feedback on the enactment of employees’ creative ideas?

The remainder of the present paper is arranged as follows. In Section 2, we mainly elaborate on the hypothesis development. Section 3 primarily describes the method, while Section 4 presents the results of this article. Lastly, Section 5 offers the discussion, including conclusions, theoretical implications, practical implications, limitations, and future research.

## Hypothesis Development

### Supervisor Developmental Feedback and the Enactment of Employees’ Creative Ideas

Supervisor developmental feedback is the extent to which supervisors promote employees’ learning, development, and improvement by providing valuable information to employees (Zhou, 2003). According to the research of Zhou (2003), supervisor developmental feedback holds three distinctive features. First, it belongs to information-based feedback rather than control-based feedback, because it provides employees with valuable and beneficial information. Consequently, it cultivates a creativity-supportive content, in which there is no mandatory requirement for employees’ goals at work. Second, it has a future-oriented attribute, aiming to help employees learn, develop, and make improvements. Third, although supervisor developmental feedback implies both positive feedback information and negative evaluation information, it focuses on the transmission of information related to task improvement rather than the positive and negative feedback valence. These characteristics reflect the widespread functionality and universal importance of supervisor developmental feedback in organizational activities.

Research on social exchange theory and Pygmalion Effect provides a direct basis for understanding how supervisor developmental feedback facilitates the enactment of employees’ creative ideas. Social exchange theory is widely used in the field of social psychology, which means that human behavior is dominated by a certain kind of exchange activity that can bring rewards (Cropanzano et al., 2017; Montani et al., 2017). The exchange relationship between supervisors and employees would lead to high-level employee innovative behavior. The emergence of supervisor developmental feedback creates a situation where the exchange party provides constructive resources. To maintain and strengthen such social exchange relationship, employees devote more energy and time to respond to the supervisors’ feedback, thereby enhancing employees’ intrinsic motivation to exceed the basic requirements stipulated in their contract (Li et al., 2011; Eva et al., 2019; Su et al., 2019). Meanwhile, the Pygmalion Effect suggested that positive external expectations improved individual performance (Tierney and Farmer, 2004). Supervisor developmental feedback reflects the supervisor’s expectations and commitment to employees’ development and personalization. According to the principle of reciprocity, employees understand supervisor developmental feedback broadly within or outside the scope of the task, and could maintain cognitive vigilance, modify practical actions when necessary, seek opportunities to improve technical methods, and even exceed work requirements.

Through the above comprehensive analysis, we contend that supervisor developmental feedback plays a crucial role in persuading employees to enact creative ideas. First, exposure to developmental and surprising feedback is conducive to cultivating employees’ divergent thinking as a way of reciprocating for the positive treatment they have received from the supervisors (Zhang et al., 2017). Then, the harmonious team climate created by supervisor developmental feedback strengthens employees’ psychological safety. This could fundamentally reduce employees’ fear of engaging in transformational work and help them form positive expressions of creative ideas (Wu and Parker, 2017; Tu et al., 2019). Finally, from the perspective of work dynamics, supervisor developmental feedback could further unlock information resources, which makes employees not only limited to the generation of creative ideas (Zhang et al., 2019), but also helps employees show the feasibility and potential value of their creative ideas in the form of physical carriers. Therefore, the following hypothesis was proposed:

*Hypothesis* 1: Supervisor developmental feedback relates positively to the enactment of employees’ creative ideas.

### Mediating Influence of Positive Emotions

The feedback employees receive from their supervisors are intuitively mirrored in their emotions and professional practices. Positive emotion is a short-lived positive evaluative status, with cognitive and neurological elements. The specific aspects to which employees respond to positive emotions are recognition and achievement (Tepper et al., 2018), while such events are more likely to arise in a resourceful job, with a high level of task significance, autonomy, and feedback (Nifadkar et al., 2012). The essence of supervisor developmental feedback is to express the boss’s concern about the work and needs of followers, aiming to enhance subordinates’ positive emotions by improving subordinates’ happiness and work enthusiasm. For example, Zhang et al. (2019) hold that supervisor developmental feedback signals to employees that their supervisors support and cherish the benefits they bring to the company, and discover a positive relationship between day-level supervisor developmental feedback and day-level positive affect. Hence, supervisor developmental feedback would induce employees’ positive emotions.

Positive emotions are expected to facilitate the enactment of employees’ creative ideas. Emotions are essentially a synthesis of physiology, psychology, and sensation (Mann, 1999), and if employees share positive emotions related to innovation, they are likely to develop a favorable motivational orientation toward it and behavioral readiness for implementing it. Studies have shown that employees in a positive mood display fluid ideation, divergent thinking, flexible categorization, and perform well on perceptive problems, unusual word associations, and heuristic problem-solving tasks (George and Zhou, 2007; Torres et al., 2019). Emotion regulation strategies that belong to the category of positive emotions (Denier et al., 2020) help irritate the individual’s creative development. Further, BBT helps to understand the influence of positive emotions on employees’ creative idea enactment (Fredrickson, 1998). According to this view, positive emotions not only encourage individuals to build up personal resources such as knowledge and social support, but also enable individuals to perform more creative behaviors by expanding cognition and action range. In general, these statements suggest that positive emotions incite employees’ creative idea enactment because positive emotions have the function of affecting information processing and working memory.

Affective events theory points out that there is a complete mechanism chain of “events-emotions-attitude and behavior” in the organization. Namely, events in the workplace indirectly affect employees’ work attitudes and behaviors by triggering different emotional reactions (Weiss and Cropanzano, 1996). Existing research has already used this theoretical mechanism to investigate how authentic leadership could facilitate employees’ creativity by stimulating employees’ positive emotions (Rego et al., 2014). Therefore, we believe that supervisor developmental feedback, as a momentous event in the workplace, tends to change the mental state of employees when stimulating employees to enact ideas, that is, to boost their positive emotions at work. Then, we offered the following assumption:

*Hypothesis* 2: Positive emotions play a mediating role between supervisor developmental feedback and employees’ creative idea enactment.

### Mediating Influence of Work Engagement

Theory of Planned Behavior and the Job Demands-Resources Model highlights that feedback is one of the considerable factors that affect employee performance. Theoretically, the higher the degree to which a person is identified psychologically with his work, the more work he participates in (Lesener et al., 2019). However, this closely relies on an ecosystem of external feedback from various sources, such as from supervisors, coworkers, or critical clients (Schaufeli et al., 2002). The Theory of Planned Behavior reminds us that the employees’ organizational behavior depends on their attitude and perception of the surrounding environment (Arain et al., 2020). Supervisor developmental feedback conveys constructive signals for the development of the company to employees (Zhou and George, 2001), and in such a supportive environment, employees have the ability to master detailed information of job responsibilities, reduce ambiguity and uncertainty by clarifying performance standards, thereby increasing their level of work engagement (Eva et al., 2019). Besides, the Job Demands-Resources Model proposed that capturing and storing resources, such as training, rewards, and feedback are requisite conditions for employees to devote to their work (Bakker and Demerouti, 2017; Knight et al., 2017; Wingerden et al., 2017; Radic et al., 2020). Once these resources are provided, work engagement is more likely to occur. Following this logic, as a form of constructive feedback, supervisor developmental feedback is helpful to actively guide subordinates to recognize goals, correct deviations, improve methods, and enhance professional engagement (Menguc et al., 2013). Thus, supervisor developmental feedback is presumed to positively affect work engagement.

Besides, we posit that a high level of work engagement would stimulate the enactment of creative ideas. There are three reasons. Firstly, employees immersed in the job held higher vitality, engendering plenty of energy and good mental toughness (Black et al., 2017). Vitality is a positive emotion related to work, which promotes people to discover and generate original ideas (Kwon and Kim, 2020), thereby indirectly increasing the possibility of enacting ideas. Secondly, creative cognition needs to integrate three processes of problem identification, information search, and creativity generation (Biennewies and Gromer, 2012), while dedicated employees have a strong sense of responsibility and initiative to identify problems and search for information more effectively at work, leading to propose creative solutions (Bhatnagar, 2012). Finally, in addition to devoting themselves to existing jobs, dedicated employees undertake diversified advantages, such as withstanding more failures, having mental resilience to generate novel ideas, and seeking opportunities to excel (Orth and Volmer, 2017). These diversified advantages help employees increase their determination to overcome difficulties and enhance the possibility and strategic fit of enacting ideas. To sum up, it is plausible to predict that supervisor developmental feedback is assumed to positively affect creative idea enactment by increasing employees’ work engagement. Therefore, the following assumption was put forward:

*Hypothesis* 3: Work engagement plays a mediating role between supervisor developmental feedback and the enactment of employees’ creative ideas.

### The Chained Mediating Effect of Positive Emotion and Work Engagement

Positive emotions are expected to initiate employees in being engaged in the work, and we thus strive to integrate positive emotions and work engagement into a broader framework. To accomplish this integration, we have synthesized relevant literature and applied BBT. Work engagement is described as a motivational concept that is associated with the fulfillment of personal aspirations (Schaufeli et al., 2002; Schaufeli and Bakker, 2010). In the literature, employees’ psychological state, including emotions (such as high energy, excitement, pleasure, pride, etc.) caused by workplace events has a positive function on work completion (Conner and Silvia, 2015). The uniqueness of BBT lies in the introduction of two critical assumptions, “Broaden hypothesis” and “Build hypothesis.” “Broaden hypothesis” argues that positive emotions enable an individual to tap into a wider range of thoughts by momentarily expanding one’s thinking and attention. In turn, “Build hypothesis” points out that these broadened outlooks may challenge difficult problems by establishing consequential personal resources, and enable individuals to obtain happiness and success from them (Ouweneel et al., 2012). In other words, positive emotions are proven to expand people’s thought-action combinations and to “build” personal resources, like hope, which in turn lead to a state of well-being, like participation (Buric and Macuka, 2018). Evidence has shown that employees who experience positive emotions are full of enthusiasm, vigor, and courage to devote extra effort to the job (Gloria and Steinhardt, 2017; Young et al., 2018), because they exhibit higher levels of resilience and fewer symptoms of burnout.

Upon the analysis above, we propose a chain mediation effect between supervisor developmental feedback and the enactment of employees’ creative ideas. Namely, supervisor developmental feedback could increase employees’ positive emotions, and positive emotions are positively related to work engagement by broadening one’s thinking and accumulating more resources, ultimately facilitating vivid presentation of creative ideas. Therefore, the following assumption was made:

*Hypothesis* 4: Positive emotions and work engagement play a chain mediating role in the relationship between supervisor developmental feedback and employees’ creative idea enactment.

### The Moderating Role of Psychological Empowerment

The intensity of positive emotion that an individual perceives from feedback is subject to the interference of individual characteristics. The AET states that employees’ emotional perception in the workplace may be influenced by their characteristics, while psychological empowerment refers to people’s various psychological states stemming from environmental differences, which affect individual characteristics (Smith et al., 2019). Specifically, psychological empowerment consists of four core cognitions of work: competence, meaning, self-determination, and impact (Spreitzer, 1995; Matsuo, 2021). Previous research has generally linked psychological empowerment to significant employee outcomes in the team (Hartmann et al., 2018; Garcia-Juan et al., 2020). Schermuly and Meyer (2016) proposed that there was a negative correlation between psychological empowerment and feelings of depression. They also mentioned that accepting positive feedback stimulated the competence dimension of psychological empowerment, thereby allowing individuals to gain more resources to feel more impactful. Thus, we argue that psychological empowerment should not be isolated from feedback research.

The effectiveness of the supervisor developmental feedback is a dynamic process, where the feedback process needs to fit subordinates and the environment to achieve effective results. Psychological empowerment makes employees feel more “empowered” and motivates them to change their inner beliefs and attitudes (Spreitzer, 1995). Supervisor developmental feedback provides a creativity-supportive context that enables individuals to generate better creative solutions, which is in line with the characteristics of highly empowered employees, that is, they value organizational goals and implement positive behaviors. When employees’ psychological empowerment is high, employees have sufficient centripetal force and competitiveness to increase positive emotions at work, implement independent actions, and overcome psychological barriers to expertise seeking (Hong and Gajendran, 2018; Garcia-Juan et al., 2020). As a result, such employees perceive weaker positive emotions from supervisor developmental feedback. Conversely, employees with low psychological empowerment lack confidence to complete tasks and the internal drive to work, thus they perceive stronger positive emotions from supervisor developmental feedback, and believe that they can change the working environment. In contrast, supervisor developmental feedback is more effective in motivating followers with low psychological empowerment, because these followers pay more attention to the acquisition of professional knowledge and resources (Chen et al., 2019). Therefore, this study considers psychological empowerment as a moderator and presumes that the positive effect of supervisor developmental feedback on positive emotions would be further weakened by psychological empowerment.

Through the above theoretical derivation, we conjecture that highly empowered employees perceive higher work meaning and self-determination. These advantages of highly empowered employees greatly decrease the extent to which individuals obtain positive emotional experience from supervisor developmental feedback, thereby reducing the indirect influence of supervisor developmental feedback on creative idea enactment through positive emotions and work engagement. Thus, we developed the following hypothesis:

*Hypothesis* 5: Psychological empowerment negatively moderates the relationship between supervisor developmental feedback and positive emotions.
*Hypothesis* 6: Psychological empowerment negatively moderates the chain mediation effect of positive emotions and work engagement between supervisor developmental feedback and the enactment of employees’ creative ideas.

**Figure 1:**
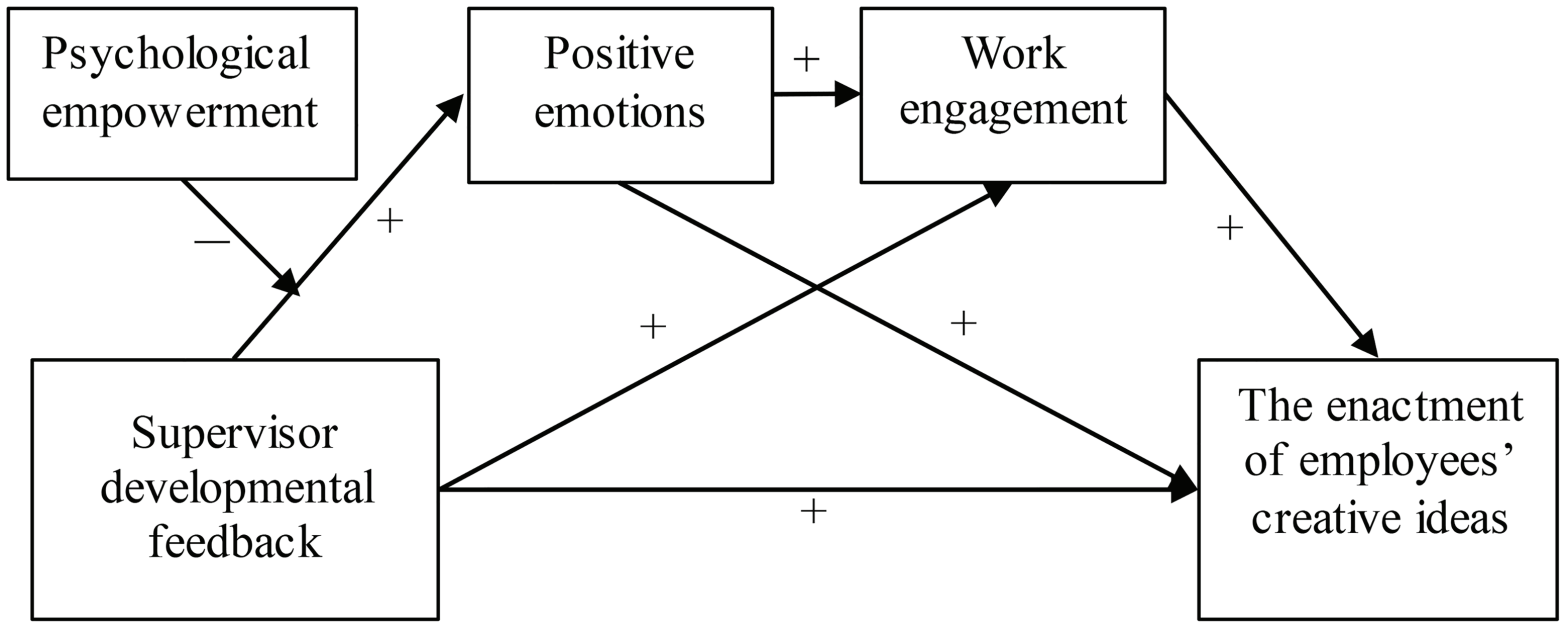
The hypothesized model.

Our research model is captured in Figure 1.

## Materials And Methods

### Research Methods

Since questionnaire survey is a relatively popular research technique, which has been widely used in studies of different industries and diverse fields (Zhong et al., 2018; Belad and Joumni, 2020; Zyl et al., 2021), this article adopted the method of a questionnaire survey to collect the original data. Subsequently, we conducted a quantitative study, listing descriptive statistical analysis and hierarchical regression analysis in turn. During the questionnaire survey, online data collection was applied for two major reasons, first, it holds various advantages, such as high quality, low cost, and high speed (Rasool et al., 2021). For example, respondents are more likely to make true answers to privacy and sensitive questions, and information transmission and feedback are both faster and more efficient. Second, the data collection in this article was carried out during the COVID-19 pandemic, when a majority of employees were telecommuting or working from home. Refer to the research of Samma et al. (2020) and Rasool et al. (2021); this article explains in detail the questionnaire development, samples, and data collection.

### Questionnaire Development

To measure variables reliably and effectively, a questionnaire comprised of 40 items was preliminarily designed according to classical studies in related fields. All variables were measured using a five-point Likert scale where “1” meant strongly disagree and “5” meant strongly agree, and respondents were invited to rate the statement from strongly disagree to strongly agree. Consistent with previous research (Liang et al., 2019), all items were translated from English to Chinese following the translation/back-translation procedure. Notably, a pilot investigation was performed to verify the reliability and validity of the instrument: We distributed questionnaires to 10 professors in related fields and 10 corporate managers (who have extensive organizational management experience and are familiar with this research topic) to obtain feedback on the instrumental design. It ultimately led to several minor changes in the item wording and language logic. In a word, the questionnaire survey of this article was logically clear and feasible, and was described in detail in Appendix A.

### Sample

The sample of this survey comprised six emerging companies located in Shaanxi and Henan provinces, China, and these companies cover diverse industries with high demand for innovation, including communications, healthcare, and the Internet. The reason for choosing companies in these two cities is that these enterprises are easy to get in touch and maintain communication through social relations, and the contact information of the target interviewees obtained is true and reliable. Sample 1 (*N*=116) consisted of employees of a healthcare company. The sample contained 65 males (56 per cent) and 51 females (44 per cent). The core business of this company is the technical development, technical consulting, technical training of medical and healthcare products, as well as the development, promotion, and database maintenance of medical software. Sample 2 (*N*=78) consisted of employees of an Internet technology company. The sample included 52 males (67 per cent) and 26 females (33 per cent). Most employees have a master’s degree and the mean age is 28years. The main activities of the employees are computer integration and the development and sale of software and hardware. No redundant introduction to other samples.

### Data Collection

To ensure the quality of the data obtained, the drafted guidelines explained to the interviewees that the purpose of this survey was only for academic research, the answers could be withdrawn at any time, and anonymity and confidentiality were guaranteed at the beginning of each investigation section. In the preliminary study, we used the convenience sampling method in non-probability sampling because this method allows researchers to gain necessary data and research trends (Samma et al., 2020). Notably, before formal data collection, this paper conducted a pre-test. In this process, 80 questionnaires were obtained, and the analysis results indicated that these questionnaires passed the reliability and validity tests.

Next, we performed a two-time survey to formally collect data. Theoretically, to achieve a 95% confidence rate, the sample size should be greater than 300. Considering that there may exist invalid responses to the questionnaire, we distributed 600 questionnaires during the first survey. Specifically, at time one, employees were asked to provide demographic information and to rate the level of feedback they received from their supervisors. A total of 600 questionnaires were returned at this stage, of which 518 were valid questionnaires, resulting in an effective response rate of 86.33%. One month later, 518 respondents who responded to the first survey were asked to report their emotional state and the level of work engagement. A total of 397 valid questionnaires were obtained, with an effective response rate of 76.64%. The reason for using multi-time and multi-source data is that data from common sources may exaggerate the correlation between variables, leading to biased conclusions (Li et al., 2018; Su et al., 2019).

### Variables and Measurement

#### Supervisor Developmental Feedback

Using the scale developed by Zhou (2003) to measure supervisor developmental feedback. The measurement standard contains three items: “My immediate supervisor often gives me developmental feedback,” “The focus of feedback given to me by my superior is to help me learn and improve,” and “Supervisor feedback provides me with useful information on how to improve my work performance.” The average of these three items forms the supervisor developmental feedback scale. Lastly, the Cronbach’s alpha for this measure was 0.834, which was higher than the critical value of 0.7, indicating that the items used in our research instrument were valid.

#### Positive Emotions

Positive emotions were assessed utilizing the classic questionnaire instruments (Tellegen et al., 1999). These five items were expressed as follows: “in the past few weeks, my work had made me feel “strong, excited, interested, enthusiastic, and determined.” The Cronbach’s α for the positive emotion scale was 0.886. The items adopted in the research were considered quite valid because their Cronbach’s α value was higher than the standard 0.70.

#### Work Engagement

Work engagement was measured using the Utrecht Work Engagement Scale designed by Schaufeli et al. (2002). This scale has three dimensions and finally contains 17 items. The sample items are: “When I get up in the morning, I feel like going to work,” “I am proud of the work that I do,” “I feel happy when I am working intensely,” and so on. The Cronbach’s α for this measure was 0.910. Given that the standard value of Cronbach’s α is 0.70 or higher, we have ample reasons to believe that the items used in our instrument were valid.

#### The Enactment of Creative Ideas

Drawing on the research of Lu et al. (2019), three items were used to assess the enactment of creative ideas. This scale asks participants a question, “how frequently do you engage in the following behaviors when you try to provide a creative idea to your supervisor,” and these three behaviors are: “Illustrate your ideas to the supervisor through written descriptions, PowerPoint presentations, drawings, or storyboards,” “Conduct experimental simulations yourself to prove whether the creative ideas are feasible,” and “Develop a prototype or other sample to prove the value of the creative ideas.” The items we used in the research instrument were valid because the value of Cronbach’s α was 0.784, which was higher than the critical value of 0.7.

#### Psychological Empowerment

Based on the research of Spreitzer (1995), a translation of the entire 12-item questionnaire was used to assess psychological empowerment. The scale consists of four dimensions with three items each: meaning, competence, self-determination, and impact. Sample items are “I can decide for myself how to do my job” (self-determination, *α*=0.84), “The work I do is meaningful to me” (meaning, *α*=0.87), “I have a great deal of control over what happens in the department” (impact, *α*=0.86), and “I am confident about my ability to do my job” (competence, *α*=0.82). The Cronbach’s α for the psychological empowerment scale was 0.850. The standard value of Cronbach’s α was 0.70 or higher, thus, the items utilized in our research instrument were fairly valid.

### Participants’ Characteristics

Our questionnaire was distributed to 600 employees of SMEs, and a total of 397 valid responses were used for the analysis of this article. The socio-demographic characteristics of the participants were summarized from four aspects. In this study, 54.2% of participants were male. In terms of education, 78.6% had a bachelor’s degree or above, of which 30.5% had a master’s degree and 3.0% had a doctoral degree. Respondents with other educational backgrounds accounted for 21.4%. The age distribution of the investigators was that 16.6% of those aged 25 and under, 74.8% of those aged 26–35, 7.6% of those aged 36–45, and 1.0% of those aged 46 and over, reflecting that the investigators were highly educated and younger. There were 156 respondents with a working age of 3years and below, accounting for 39.3%, 104 of respondents had worked for 3–5years, accounting for 26.2% of the total sample, and respondents who have worked for more than 5years accounted for 34.5%. The detailed socio-demographic characteristics are shown in Table 1.

**Table 1:** The detailed socio-demographic characteristics of respondents. (*N*=397).

Characteristics	Category	Frequency (*n*)	Percentage (%)
Gender	Male	215	54.2
	Female	182	45.8
Working experience	3years and below	156	39.3
	3–5years	104	26.2
	Above 5years	137	34.5
Education	Bachelor’s degree	179	45.1
	Master’s degree	121	30.5
	Doctor’s degree	12	3.0
	Others	85	21.4
Age	25years old and below	66	16.6
	26–35years old	297	74.8
	36–45years old	30	7.6
	46years old and above	4	1.0

Following previous research (Dimotakis et al., 2017; Gloria and Steinhardt, 2017), we created a demographic similarity measure that included followers’ gender (male=1, female=0), age (25years old and below=1, 26–35years old=2, 36–45years old=3, and 46years old and above=4), work tenure (3years and less=1, 3–5years=2, 5–10years=3, and 10years or more=4), and education (1=undergraduate, 2=undergraduate, 3=master’s degree, and 4=doctoral degree) to improve the accuracy of hypothesis testing.

### Analytical Strategy

We first used the three-step test method of Baron and Kenny (1986) to shed light on the mediating role of positive emotions and work engagement in the relationship between supervisor development feedback and employees’ creative idea enactment. The specific operation was to use SPSS to perform a hierarchical regression analysis which is widely used in many quantitative studies (Peltokorpi and Hasu, 2016; Ugur et al., 2018). First, we took the supervisor developmental feedback and other control variables as a whole to perform regression (see model 5 in Table 2). Then, we included potential mediators into the model (see models 6 and 7 in Table 2). Finally, we entered independent variables and the two mediators as a block (see model 8 in Table 2). According to the research of Preacher and Hayes (2004), bootstrap methods in virtue of the PROCESS program in SPSS were used to further verify the existence of the chain mediating effect. Bootstrapping is especially advantageous in this research because indirect effects usually do not comply with a normal distribution and the sample size we used is relatively small. We bootstrapped with 5,000 in the study to produce bias-corrected CIs of yield 95%, and the chained mediating effect was significant if the CI excluded 0.

**Table 2:** Hierarchical regressions for main variables.

Variables	Positive emotions		Work engagement		Enactment of employees’ creative ideas				Model 1	Model 2	Model 3	Model 4	Model 5	Model 6	Model 7	Model 8
Gender	−0.01	−0.09	−0.02	−0.02	−0.09	−0.09	−0.08	−0.08
Age	−0.04	−0.14	−0.06	−0.05	−0.12	−0.11	−0.10	−0.10
Work tenure	0.03	0.05	0.03	0.03	0.05	0.04	0.04	0.04
Education	0.01	0.12	0.07	0.07	0.09	0.09	0.07	0.08
SDF	0.53^***^	0.41^***^	0.40^***^	0.27^***^	0.51^***^	0.37^***^	0.43^***^	0.33^***^
positive emotions				0.25^***^		0.26^***^		0.22^***^
work engagement							0.21^***^	0.16^***^
PS		−0.06^**^						
SDF×PS		−0.09^**^						
R^2^	0.28	0.30	0.18	0.22	0.29	0.34	0.33	0.36
ΔR^2^	—	0.02	—	0.04	—	0.05	0.04	0.07
F	29.1^***^	23.1^***^	16.3^***^	18.1^***^	30.8^***^	32.4^***^	30.3^***^	30.2^***^

*SDF represents supervisor developmental feedback; EECI represents enactment of employees’ creative ideas; and PS represents psychological empowerment*.

** *p<0.01*;

*** *p<0.001*.

Then, model 83 in PROCESS macro of SPSS was performed to test the moderated chain mediation model. Only when the CI does not include 0, can psychological empowerment moderate the chained mediating effect of positive emotions and work engagement between supervisor developmental feedback and employees’ creative idea enactment. All independent variables were grand-mean centered in statistical analysis.

## Results

### Validity of Scales

The present study, respectively performed tests of content validity, convergent validity, and discriminant validity. First, the measurement of main variables came from relatively mature scales in academia and was widely applied in Chinese management situations (Zhang et al., 2017; Su et al., 2019), which meant that our scale had good content validity. Secondly, we found that AVE values of the five variables of supervisor developmental feedback, positive emotions, work engagement, employees’ creative idea enactment, and psychological empowerment were 0.637, 0.602, 0.549, 0.548, and 0.660, respectively, which were greater than the critical value of 0.5, indicating that the five variables had good convergent validity. Finally, confirmatory factor analyses (CFA) were conducted by using a weighted least squares means and variance-adjusted (WLSMV) estimator in Mplus7.2, and relevant results were presented in Table 3. Compared with one-factor model, two-factor model, three-factor model, and four-factor model, the proposed five-factor model yielded a good fit to the data (χ^2^=610.864, df=289, χ^2^/df=2.114, CFI=0.956, GFI=0.902, IFI=0.956, TLI=0.910, and RMSEA=0.054), implying that respondents could distinguish all constructs clearly.

**Table 3:** The result of confirmatory factor analysis (*N*=397).

Factors	*χ* ^2^	df	*χ^2^/df*	CFI	GFI	IFI	TLI	RMSEA
Five-factor model (SDF; PE; WE; EECI; PS)	610.864	289	2.114	0.956	0.902	0.956	0.910	0.054
Four-factor model (SDF+PE; WE; EECI; PS)	756.896	293	2.583	0.937	0.858	0.937	0.964	0.064
Three-factor model (SDF+PE+WE; EECI; PS)	826.094	296	2.791	0.927	0.843	0.928	0.867	0.068
Two-factor model (SDF+PE+WE+EECI; PS)	859.223	298	2.883	0.923	0.834	0.923	0.822	0.070
Single-factor model (SDF+PE+WE+EECI+ PS)	1067.394	299	3.570	0.895	0.791	0.895	0.79	0.091

*SDF represents supervisor developmental feedback; PE represents positive emotions; WE represents work engagement; EECI represents enactment of employees’ creative ideas; and PS represents psychological empowerment*.

### Common Method Bias Checks

To address concerns over common method bias, several procedural remedies of anonymous filling, cross-temporal surveys, and the setting of mutually exclusive items were adopted in the process of data collection. However, the problem of common method bias may exist due to the single source of the questionnaire, then we conducted Harmon’s one-factor test using PSSS22.0. The result revealed that the first principal component factor before rotation explained 29.4% of the total variation, which was lower than the recommended value of 50%, further reflecting that common method variance was unlikely to be a serious problem in our data.

### Descriptive Statistics and Correlations Among Variables

Table 4 displayed the means, SDs, and correlations of all variables. According to the table, the mean value of SDF is 2.94, with a SD of 0.887, indicating that the dispersion of supervisor developmental feedback is large. The average value of EECI is 3.17, with a SD of 0.893, which demonstrates that the level of creative idea enactment is relatively high. An inspection of the correlations showed that supervisor developmental feedback had a significant positive impact on positive emotion (*γ*=0.411, *p*<0.001), work engagement (*γ*=0.436, *p*<0.001), and the enactment of employees’ creative ideas (*γ*=0.449, *p*<0.001). Meanwhile, positive emotions (*γ*=0.429, *p*<0.001) and work engagement (*γ*=0.369, *p*<0.001) were positively related to the enactment of creative ideas, respectively. Besides, there was a significant positive correlation between positive emotions and work engagement (*γ*=0.452, *p*<0.001). Hence, the results of correlation analysis were consistent with our theoretical expectations, then, subsequent hierarchical regression analysis can be promoted.

**Table 4:** Descriptive analysis and correlations among variables.

S.No	Variables	1	2	3	4	5	6	7	8	9
1	Gender	1								
2	Age	−0.004	1							
3	Work tenure	0.002	0.314^**^	1						
4	Education	0.104^*^	0.282^*^	0.166^*^	1					
5	SDF	−0.089	0.044	0.039	0.106^*^	1				
6	Positive emotions	−0.084	−0.006	0.004	0.035	0.411^**^	1			
7	Work engagement	−0.068	0.012	0.008	0.047	0.436^**^	0.452^**^	1		
8	EECI	−0.103^*^	0.015	0.020	0.098	0.449^**^	0.429^**^	0.369^**^	1	
9	PS	−0.086	−0.017	−0.031	0.043	0.363^**^	0.344^**^	0.441^**^	0.466^**^	1
Mean		0.54	2.51	2.07	2.18	2.94	3.03	3.18	3.17	3.24
SD		0.496	0.517	1.059	0.779	0.887	0.775	0.829	0.893	0.951

*SDF represents supervisor developmental feedback; EECI represents enactment of employees’ creative ideas; and PS represents psychological empowerment*.

* *p<0.05*;

** *p<0.01*.

### Hypotheses Testing

Table 2 provided the results of hierarchical regression analysis. Hypothesis 1 predicted a positively direct effect of supervisor developmental feedback on the enactment of employees’ creative ideas. Model 5 of Table 2 showed that supervisor developmental feedback was significantly related to employees’ creative idea enactment (*β*=0.51, *p*<0.001), thus supporting Hypothesis 1.

Our paper adopted the three-step procedure of Baron and Kenny (1986) for justifying the mediation effect. Firstly, supervisor developmental feedback should be significantly associated with the enactment of employees’ creative ideas. Secondly, after adding mediating variables, the association between mediating variables and creative idea enactment should be significant. Finally, the indirect impact of supervisor developmental feedback on employees’ creative idea enactment must be significant as well. As model 6 of Table 2 showed, when adding positive emotions to model 5, it significantly predicted the enactment of employees’ creative ideas (*β*=0.26, *p*<0.001), meanwhile, the effect of supervisor developmental feedback on employees’ creative idea enactment was still significant (*β*=0.37, *p*<0.001). Hence, positive emotions partially mediated the influence of supervisor developmental feedback on the enactment of employees’ creative ideas, supporting Hypothesis 2. Similarly, Model 7 in Table 2 stated that there was a significant indirect effect for work engagement in the relationship between supervisor developmental feedback and creative idea enactment. Hence, Hypothesis 3 was supported. Furthermore, supervisor developmental feedback, positive emotion, and work engagement were included in the regression model at the same time, and it was found that all three of them still had a significant positive effect on the enactment of employees’ creative ideas (*β*=0.33, *p*<0.001; *β*=0.22, *p*<0.001; *β*=0.16, *p*<0.001). Hypothesis 4 was preliminarily supported.

Using a bootstrap estimation procedure to confirm the significance of mediating effect and the chained mediating effect. The results were shown in Table 5. As Table 5 presented, supervisor developmental feedback had a significant indirect effect on the enactment of creative ideas *via* positive emotions, and the 95% CI excluded zero [index=0.1178, CI=(0.0539, 0.1882)]. The mediating effect of work engagement was significant as well, and zero was not contained in the 95% CIs [index=0.0426, CI=(0.0152, 0.0837)]. Meanwhile, positive emotions and work engagement had a significant chained mediating effect between supervisor developmental feedback and employees’ creative idea enactment, with the 95% CI excluded zero [index=0.0213, CI=(0.0073, 0.0438)]. Therefore, Hypothesis 2, Hypothesis 3, and Hypothesis 4 were confirmed again. Moreover, the single-mediation effect of positive emotion was stronger than the single-mediation effect of work engagement and the chained mediating effect, while the difference between the single-mediation effect of work engagement and the chained mediating effect was not significant [CI=(−0.0602, 0.0006)].

**Table 5:** Conditional indirect effects of the chained mediation analyses.

Path	Indirect effects	Boot SE	95% CIs	Percentage of total effect
Ind1: SDF-PE-EECI	0.1178	0.0339	(0.0539, 0.1882)	23.14%
Ind2: SDF-WE-EECI	0.0426	0.0171	(0.0152, 0.0837)	8.34%
Ind3: SDF-PE-WE-EECI	0.0213	0.0091	(0.0073, 0.0438)	4.17%
Ind1-Ind2	0.0751	0.0403	(0.0008, 0.1595)	-
Ind1-Ind3	0.0965	0.0373	(0.0266, 0.1739)	-
Ind2-Ind3	0.0214	0.0149	(−0.0602, 0.0006)	-

*SDF represents supervisor developmental feedback; PE represents positive emotions; WE represents work engagement; and EECI represents enactment of employees’ creative ideas*.

**Figure 2:**
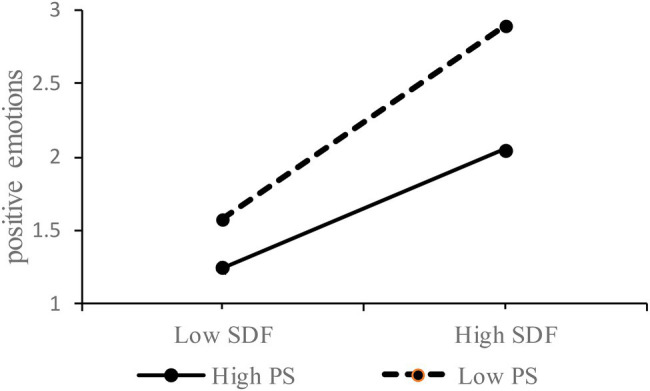
The moderating effect of psychological empowerment on the influence of supervisor developmental feedback on positive emotions. SDF represents supervisor developmental feedback; PS represents psychological empowerment.

Next, Model 2 in Table 2 presented that the interaction term for supervisor developmental feedback and psychological empowerment was significant in predicting positive emotions (*β*=−0.09, *p*<0.01). Hence, psychological empowerment had a negative moderation effect on the relationship between supervisor developmental feedback and positive emotions, supporting Hypothesis 5. Figure 2 illustrated the nature of the interaction term by using simple slopes for high and low values of psychological empowerment (mean value plus/minus one SD). As saw in Figure 2, the relationship between supervisor developmental feedback and positive emotions was stronger when psychological empowerment was low and weaker yet still positive when psychological empowerment was high.

The results of the moderated chained mediating effect obtained by running PROCESS macro were shown in Table 6. The moderated effect was shown at 1SD above the mean, the mean, and 1SD below the mean. The results displayed that when psychological empowerment took a low value, the mediating effect of supervisor developmental feedback on employee’s creative idea enactment through positive emotions and work engagement was 0.0331, and the 95% CI excluded zero [index=0.0331, CI=(0.0163, 0.0538)]. When psychological empowerment took a high value, the mediating effect of supervisor developmental feedback on the enactment of employee’s creative idea was reduced to 0.0162, and the 95 percent CI did not contain zero [index=0.0162, CI=(0.0043, 0.0390)]. The difference between the chain mediation effect value when psychological empowerment was high and the chain mediation effect value when psychological empowerment level was low was −0.0169, with the CI did not contain zero [CI=(0.0021, 0.0340)]. Taken together, psychological empowerment negatively moderates the chained mediating effect of positive emotions and work engagement between supervisor developmental feedback and the enactment of employees’ creative ideas. Hypothesis 6 was fully supported.

**Table 6:** Indirect effects of the moderated chained mediation analyses.

	Indirect effects	Boot SE	95% CIs
−1SD below the mean	0.0331	0.0348	(0.0163, 0.0538)
Mean	0.0246	0.0293	(0.1042, 0.2209)
+1SD above the mean	0.0162	0.0311	(0.0043, 0.0390)

## Discussion

Supervisor developmental feedback and employee innovation have attracted the attention of individual researchers. Nevertheless, scant quantitative studies pay attention to the enactment of employees’ creative ideas. This is the first study to examine the influence of supervisor developmental feedback on the enactment of employees’ creative ideas, and is also the first study to link feedback with the enactment of ideas through employees’ psychological feelings and workplace attitudes.

First, the research results show that there is a positive correlation between supervisor developmental feedback and the enactment of employees’ creative ideas. This supports Hypothesis 1. Although the result has not been confirmed in previous studies, several related studies furnish a strong logical basis for it. That is, Li et al. (2021) accentuated that supervisor developmental feedback facilitates employees’ innovative behavior, while Harvey (2014) highlighted that the enactment of innovative ideas is considered a pivotal part of employees’ creative process. This finding is also supported by social exchange theory (Cropanzano et al., 2017). Once the supervisor developmental feedback is accepted by the employees, it is easier for these recipients to find and solve the problems at work.

Secondly, the outcomes of this paper point out that positive emotions play a mediating role between supervisor developmental feedback and the enactment of employees’ creative ideas. Hypothesis 2 is thus verified. This result fits well with the AET, that is, organization members have emotional experiences in the events experienced in the workplace, and in turn, the emotions perceived by employees affect their attitudes and behaviors (Madrid et al., 2019). Meanwhile, BBT also supports this result, which corroborates an intuition that in a highly uncertain and innovative environment, employees with positive emotions have the confidence to find creative approaches (Amabile et al., 2005).

Thirdly, the findings also indicate that work engagement plays mediating role between supervisor developmental feedback and employees’ creative idea enactment. Hypothesis 3 is confirmed. Previous studies have similar confirmations. For instance, Eva et al. (2019) emphasized that supervisor feedback stimulates employees’ innovative behaviors by reducing employees’ sense of psychological contract violation or enhancing employees’ work engagement. Based on the Theory of Planned Behavior and the Job Demands-Resources Model, Afsar et al. (2020) and Mubarak et al. (2021) demonstrated that work engagement is a pivotal catalyzing factor in boosting innovative behavior.

Fourthly, the present paper confirms the chained mediating role of positive emotions and work engagement in the relationship between supervisor developmental feedback and employees’ creative idea enactment, which supports Hypothesis 4. This endorsed the argument of Wu and Wu (2019) that positive emotion is a psychological state generated by emotional changes, which could boost the innovation behavior by increasing employees’ work engagement.

Finally, our results reveal that psychological empowerment not only negatively moderates the relationship between supervisor developmental feedback and positive emotions, but also negatively moderates the chained mediating effect of positive emotions and work engagement. Both Hypothesis 5 and Hypothesis 6 are established. This result is in line with the logic of the AET, namely, the intensity of emotional response that employees perceive from workplace events is interfered by individual characteristics which of course also contains the degree of individual psychological empowerment.

### Conclusion

The conclusions of this article are as follows: First, the presented model acknowledges that supervisor developmental feedback relates positively to employees’ creative idea enactment. As expected, employees who receive developmental feedback from their superiors are motivated to demonstrate abstract creative ideas through animations or other physical objects (models, drawings, and posters) to reduce the uncertainty of these ideas and enhance mutual identification with feedback providers. In addition, supervisor developmental feedback stimulates the enactment of creative ideas by enhancing employees’ positive emotions or work engagement. Simultaneously, positive emotions and work engagement form a chained mediation between supervisor developmental feedback and employees’ creative idea enactment. The unique learning and future orientation of developmental feedback effectively regulate the internal motivation of employees and enhance the positive feelings and enthusiasm of subordinates to change the status quo, thereby facilitating employees to depict the specific form, applicability, and function of innovative ideas through visual or physical support. Lastly, psychological empowerment weakens the positive impact of supervisor developmental feedback on employees’ positive emotions, and the higher the level of psychological empowerment, the weaker the indirect effect of supervisor developmental feedback on the enactment of creative ideas through positive emotions and work engagement. It is enlightening that psychological empowerment is salient in changing individuals’ emotional responses to the same stimuli.

### Theoretical Implications

The empirical analysis provided four meaningful theoretical implications. The first contribution is dedicated to enriching innovation and HRM literature by integrating valuable findings regarding the relationship between supervisor developmental feedback and creative idea enactment into a coherent theoretical model. Most prior studies about HRM focused on organizational commitment, job satisfaction, and public service motivation (Bakker and Demerouti, 2017), relatively few studies investigated the enactment of creative ideas. Meanwhile, in contrast to the broad concept of employee creativity, creative idea enactment is easier to attract the assessment of managers and demonstrates the specificity, novelty, and value of ideas (Lu et al., 2019). Hence, this study investigated the role of supervisor developmental feedback in facilitating employees’ creative idea enactment for the first time. Some innovation literature has witnessed the theoretical development of the relationship between supervisor developmental feedback and organizational innovation (Su et al., 2019; Li et al., 2021), which provides a logical basis for our study’s theoretical derivation. In short, the study helped to shift the dominant focus in the creativity literature from idea generation to idea enactment because enacting ideas was a momentous yet neglected question that offered meaningful insights above and beyond the generation of creative ideas.

Our second contribution attempts to extend the research on the antecedent variables of positive emotions and work engagement by explaining the influence mechanism of supervisor developmental feedback on employees’ creative idea enactment. Recently, Su et al. (2019) pointed out that a key mediating mechanism through which supervisor developmental feedback was associated with employee’s innovative behavior was employee’s creative self-efficacy. Bak (2020) noted that trust in supervisors mediated the effect of supervisor feedback on innovative work behavior. However, the underlying psychological mechanism explaining the connection between supervisor feedback and creative idea enactment has not been tested in previous studies. Addressing this limitation, the present study used AET to reveal the mediating role of positive emotions between supervisor developmental feedback and the enactment of creative ideas. This deepened the understanding of the antecedents of employees’ creative idea enactment from a psychological level. Similarly, leveraging the theory of planned behavior and the JD-R model, we developed a conceptual framework that related supervisor developmental feedback, work engagement (the psychological recognition of work), and the enactment of creative ideas. It is no doubt that our final findings enrich the relevant literature on work engagement (Eva et al., 2019).

The third contribution of this paper was to demonstrate the complex structure and dynamic mechanism between supervisor developmental feedback and employees’ creative idea enactment by constructing a chained mediation model. Departing from the previous focus on the analysis of a single intermediary variable (Eva et al., 2019; Xu et al., 2019), this article highlighted the multi-level dynamic variation of innovation in organizations, and built an in-depth transmission mechanism of “supervisor developmental feedback-positive emotions-work engagement-enactment of employees’ creative ideas.” This was a momentous yet neglected phenomenon. Previous research did not consider work engagement as a possible mechanism for positive emotions to influence innovation. From a theoretical standpoint, individuals who have experienced more positive emotions consequently achieve enhanced adaptation to stressful situations and enhance their work engagement (Gloria and Steinhardt, 2017; Young et al., 2018). Therefore, this article expanded the research on the catalytic factors of idea enactment by constructing a chain mediation model.

The fourth contribution came from the exploratory examination of the boundary conditions between supervisor developmental feedback and positive emotions. There have been pieces of research on the main-effect relationship between feedback and emotions (Zhang et al., 2019), paying inadequate attention on the corresponding boundary conditions. To make up for the deficiencies of these studies, we conjectured that employees exerted positive emotions toward the organizations in response to favorable treatment from their supervisor, whereas this process differed depending on the level of employee’s psychological empowerment. Then, we proposed a framework that incorporated the interactions between psychological empowerment and supervisor developmental feedback, both of which should be well-coordinated to fuel the enactment of creative ideas. Therefore, by introducing a moderating variable, this study enriched the literature related to psychological empowerment.

### Practical Implications

Combining the main research conclusions, several management practice enlightenments are put forward.

First, to improve the opportunities for employees to enact creative solutions, corresponding organizational system settings should be implemented in the management process, namely, the supervisor regularly provides developmental feedback to employees. Scholars have recommended that organizations ought to implement training programs to instill in leaders the ability to execute developmental feedback. Meanwhile, organizations must pay heightened attention to the quality of the supervisor’s feedback content. Followers would take innovative steps only when supervisors learn to calibrate their feedback instructions in a way that meets their subordinates’ needs.

Secondly, cultivating a positive emotional atmosphere and enhancing employees’ enthusiasm for work should not be ignored by organizations since it is not comprehensive to solely rely on the effect of supervisor developmental feedback to impel the enactment of creative ideas. Organizations may benefit by establishing a workplace that enhances employees’ positive emotions and work enthusiasm. In the context of Chinese organizations, supervisors have paid uneven attention to various work resources or personal emotions, and they do not pay enough attention to employee voice, physical health, and mental state. Therefore, fostering employees’ positive emotions to enact their creative ideas must be included in the organization’s agenda. Positive emotions could be stimulated by strengthening cultural construction or creating a relaxed, free, happy, and confident emotional atmosphere within the organization. In a similar vein, idea enactment is difficult to happen unless the organization’s employees are actively engaged in work and have the responsibility to quickly incubate or nourish these ideas (Bhatnagar, 2012; Orth and Volmer, 2017). In such, organizations are encouraged to make employees understand the value of individuals, personal training, and promotion opportunities to enhance work enthusiasm and loyalty to the collective, so as to proactively depict creative ideas.

Finally, blindly seeking to enhance employees’ level of psychological empowerment is not always conducive to the organization’s innovative activities. The establishment of an overall coordination mechanism for supervisor developmental feedback and employee’s psychological empowerment to maximize the effect of enacting creative ideas is a topic worth noting for enterprises. The results articulate that employees with low levels of psychological empowerment seem to have a heightened sensitivity to positive emotions and psychological empowerment negatively moderates the chained mediating effect of positive emotions and work engagement. As a result, managers must understand the level of followers’ psychological empowerment, which enlightens them when more attention should be paid to the balance of psychological empowerment.

### Limitations and Future Research

We acknowledge several limitations of our study that suggest directions for future research. First, the sample size we use is relatively small, which raises concerns about the robustness of our analysis results. Thus, future research could choose more representative and broader research objects. Although the results of CFA supported the construct distinctiveness of different measures, common method variance may still artificially affect the overall results (Li et al., 2018).

Another limitation has to do with the conceptualization of creative idea enactment. Our examination was rooted in a concept of idea enactment of Lu et al. (2019), which extended the idea of packaging to the creative realm. This concept has come under criticism, specifically, although it was able to gauge respondents’ willingness to implement creative ideas in the experimental studies, it is impossible to prove the actual implementation of specific projects. Future research might try to trace ideas from the proposal supported by employees, all the way to the actual implementation of the creative idea. Having said that, theorists who discussed organizational innovation proposed that innovation was a cyclic and recursive process, including the ideas’ generation, evaluation, and implementation (Harvey, 2014). Thus, a feasible solution is that future research might take time issues into account and investigate how idea enactment in the form of prototypes co-evolves over time to achieve the ultimate implementation.

The third limitation was that our study focused on a limited collection of proximal and distal consequences of supervisor developmental feedback. Further intra-individual examination of the consequences of supervisor developmental feedback needed and received is warranted. Existing research has proved that many factors could affect subordinates’ innovative behaviors, such as organizational justice, personal creativity goal, work-unit climates for innovation, or unconstrained knowledge sharing which also have been proven to affect subordinates’ innovative behaviors (Gong et al., 2017; Marshall et al., 2019; Akram et al., 2020). Hence, there is a compelling theoretical basis for developing and testing other plausible mediating models that link momentary supervisor developmental feedback and the enactment of creative ideas. Besides, feedback diversity would be high when an idea receives feedback from a diverse and heterogeneous group of supervisors (Zhu et al., 2019). We failed to analyze the influence of the supervisor’s feedback on creative idea enactment from the perspectives of the diversity of feedback or the constructive degree of the feedback, but this was a possible area for future research. Furthermore, it would be productive for future research to reveal potential boundary conditions of the influence of positive emotions on the creative idea enactment based on factors such as psychological health or team member learning.

Finally, due to the time constraints of an investigation, we are unable to measure more specific cognitive and behavioral processes and other micro-intervention mechanisms that could influence the idea enactment, which is a possible field for future research. More informal forums could be employed in the process of accelerating creative ideas, which might enhance the prospects of creative ideas or be particularly suitable in later stages of the idea execution. Despite these limitations, our paper reveals that supervisor developmental feedback has undoubtedly established a psychologically safe environment in which employees’ positive emotions and work engagement are encouraged, thereby facilitating the enactment of personal creative ideas.

## Data Availability Statement

The original contributions presented in the study are included in the article/supplementary material, further inquiries can be directed to the corresponding author.

## Ethics Statement

The study procedures were approved by the Ethics Committee of Xidian University. All procedures performed in research involving human participants were in accordance with the ethical standards of the Institutional and/or the National Research Committee.

## Author Contributions

JL was responsible for data collection. HL wrote the manuscript, designed the basic model, and analyzed the data. All authors contributed to the article and approved the submitted version.

## Funding

This work was supported by the research on the Countermeasures of Innovation and Entrepreneurship Education for College Students in Shaanxi (2016ZD06).

## Conflict of Interest

The authors declare that the research was conducted in the absence of any commercial or financial relationships that could be construed as a potential conflict of interest.

## Publisher’s Note

All claims expressed in this article are solely those of the authors and do not necessarily represent those of their affiliated organizations, or those of the publisher, the editors and the reviewers. Any product that may be evaluated in this article, or claim that may be made by its manufacturer, is not guaranteed or endorsed by the publisher.

## Figures and Tables

**Table:** 

	Supervisor developmental feedback
1.	My immediate supervisor often gives me developmental feedback.
2.	The focus of feedback given to me by my superior is to help me learn and improve.
3.	Supervisor feedback provides me with useful information on how to improve my work performance.
	Positive emotions
4.	My job made me feel strong.
5.	My job made me feel excited.
6.	My job made me feel interested.
7.	My job made me feel enthusiastic.
8.	My job made me feel determined.
	Work engagement
9.	When I get up in the morning, I feel like going to work.
10.	At my work, I feel energetic.
11.	At my work, I always persevere, even when things do not go well.
12.	I can work continuously for very long periods at a time.
13.	At my job, I am very resilient, mentally.
14.	At my job, I feel strong and vigorous.
15.	For me, my job is very challenging.
16.	My job inspires me.
17.	I am enthusiastic about my job.
18.	I am proud of the work that I do.
19.	I find that the work I do is full of meaning and purpose.
20.	When I am working, I forget everything else around me.
21.	Time flies when I am working.
22.	I get carried away when I am working.
23.	It is difficult to detach myself from my job.
24.	I am immersed in my work.
25.	I feel happy when I am working nervously.
	The enactment of creative ideas
26.	I frequently illustrate my ideas to the supervisor through written descriptions, PowerPoint presentations, drawings or storyboards.
27.	I tend to perform experimental simulations to verify the feasibility of the creative ideas.
28.	I develop a prototype or other sample to prove the value of the creative ideas.
	Psychological empowerment
29.	The work I do is very important to me.
30.	My job activities are personally meaningful to me.
31.	The work I do is meaningful to me.
32.	I am confident about my ability to do my job.
33.	I am self-assured about my capabilities to perform my work.
34.	I have mastered the skills necessary for work.
35.	I have a lot of autonomy to decide how to do my job.
36.	I can decide on my own how to go about doing my work.
37.	I have considerable opportunity for independence and freedom in how I do my job.
38.	My impact on what happens in my department is large.
49.	I have a great deal of control over what happens in my department.
40.	I have significant influence over what happens in my department.

## Appendix A

Research instrument.
